# Hospital-at-Home for Alcohol and Substance Use Disorders Compared to Inpatient Treatment in Dual Diagnosis Patients: A Retrospectively Matched Cohort Pilot Study Incorporating Service Use 12 months Pre- and Post-Treatment in Geneva

**DOI:** 10.1177/21501319251412650

**Published:** 2026-03-31

**Authors:** Silke Bachmann, Pantelis Baniotopoulos, Corinne Amoros, Louise E. Penzenstadler, Daniele F. Zullino

**Affiliations:** 1 Geneva University Hospitals, Department of Psychiatry, Addiction Services, Geneva, Switzerland; 2 University Hospitals Halle, Department of Psychiatry, Psychotherapy and Psychosomatics, Halle (Saale), Germany

**Keywords:** home treatment, alcohol use disorder, substance use disorder, dual diagnosis, addiction/addictive behaviors, 1-year follow-up, community health, program evaluation, feasibility, safety

## Abstract

**Objectives:**

This quasi-experimental study investigates the effects of the first-ever home treatment (HT), equivalent to inpatient care, for individuals with dual diagnoses: severe alcohol and/or substance use disorder plus major psychiatric illness. Outcomes are compared to those of regular inpatient treatment (IT) of the same addiction service. The primary objective was to evaluate feasibility and safety of HT. Secondary outcomes measures included discontinuation of treatment and service utilization during the 12-month follow-up period as defined by the number of emergency department visits and hospitalization days.

**Methods:**

Our Geneva model was introduced to meet local needs. In 2023, 39 individuals received home treatment (HT) for the first time, either in their own homes or in residential settings. They were retrospectively compared to a group of individuals who had undergone regular IT. Matching was based on age and gender. Allocation to IT or HT was determined by individual preferences as well as predefined inclusion and exclusion criteria following an evaluation interview. Electronic patient records were reviewed 1 year later to collect data on service use.

**Results:**

The primary outcome criteria were met. Treatment withdrawal occurred among IT patients only. At 1-year follow-up, a clear difference in addiction-related hospital days emerged in favor of HT, with smaller but still favorable for HT differences for sequelae and somatic problems. At intake, the HT and IT groups differed regarding stimulant and opioid use, as well as in the presence of the exclusion criteria history of complicated withdrawal and suicidality, which were only present in the IT group. Health of the Nations Outcome Scale (HoNOS) scores at admission and discharge did not differ between groups. Sociodemographic factors showed small differences for partnership status and housing, more pronounced ones for employment, in favor of HT patients.

**Conclusions:**

This first study on HT for dual-diagnosis patients suggests that such treatment is feasible and safe, as demonstrated for HT in general psychiatry, and may offer certain advantages over inpatient care. Key limitations include the lack of randomization, the retrospective design, limited statistical power, and the fact that data can currently only be compared with HT from general adult psychiatry.

## Introduction

### Background and Rationale

Alcohol and substance use disorders (also known as alcohol or substance use-related disorders, or AUD and SUD) represent a major public health concern. Although evidence-based treatments of people who use alcohol and/or drugs aim to provide long-term support throughout various recovery stages, certain patient groups repeatedly return to hospital services, with frequent visits to somatic emergency departments and early psychiatric readmissions.^1-3^ Numerous studies have identified the co-occurrence of AUD or SUD with other psychiatric disorders as a significant risk factor for increased healthcare utilization.^4-7^ Specifically, patients with both AUD/SUD and co-occurring psychiatric disorders show a heightened risk of frequent emergency department visits (more than 4 per year).^8,9^ Overall, up to 33% of patients with AUD/SUD are readmitted within 30 days for various medical reasons.^8,10^

Despite this, community-based treatment is less commonly available for individuals with AUD/SUD than for those with other psychiatric disorders. Certain services offered in general psychiatry are entirely lacking in addiction treatment—home treatment (HT) is one of them.

### Community and Home Treatment in General Psychiatry

In treating severe psychiatric disorders, community-based and mobile care approaches have gained increasing prominence in Europe and the United States. These approaches aim to preserve the patient’s connection to their environment, and to address issues in their natural context,^11-16^ principles that led to the development of home hospitalization or HT.^17^ HT in psychiatry shares the same goals as hospital treatment: improving physical, mental, and social health. It relocates the setting from the hospital to the patient’s home.^16^ This model inherently fosters greater interaction with patients’ families and social networks. It is offered as an equivalent alternative when hospitalization would otherwise be indicated due to an acute crisis.

The literature on HT began to expand around 2010, primarily focusing on individuals with severe mental illness characterized by persistent symptoms (lasting at least 2 years) and substantial impairments in daily functioning and social integration. These individuals frequently make extensive use of psychiatric and psychosocial services.^18^ In an early review of crisis resolution and HT teams, Hubbeling and Bertram^19^ reported that HT could reduce hospital bed use and healthcare costs while maintaining symptom control and patient satisfaction, which was unlined recently.^20-22^

Similarly, there was no increase in adverse events or compulsory hospitalizations. Other reviews found HT to be equal or superior to standard inpatient care in terms of clinical and functional outcomes, with additional benefits such as lower readmission rates, fewer treatment interruptions, higher patient and family satisfaction, and reduced caregiver burden.^23-26^

Recent research has provided robust evidence confirming HT as an effective alternative to inpatient admission. Cornelis et al^22^ presented the strongest randomized controlled trial (RCT) evidence to date, demonstrating that intensive home treatment (IHT) can safely and effectively substitute for inpatient hospitalization in acute psychiatric crises.

Bechdolf et al^20^ in a large multicenter trial, further showed that IHT led to sustained reductions in hospital bed days and readmissions over time. This trial included 400 patients, among whom 13 individuals with primary ICD-10 F1x (substance-related) diagnoses. Complementing these findings, Weinmann et al,^27^ next to premature termination, examined patient-reported outcomes such as satisfaction and shared decision-making. They found that HT fostered stronger engagement and perceived autonomy compared with inpatient care.

Collectively, these studies provide compelling evidence that IHT is a patient-centered, effective, and resource-efficient treatment modality within general psychiatry.

Multiple studies confirm that well-trained, multiprofessional teams can successfully treat acutely and severely ill psychiatric patients at home and thereby help prevent the “revolving door” phenomenon.^28^ In Switzerland, HT in general psychiatry has been associated with greater patient and family satisfaction as well as improved quality of life, appearing superior to conventional inpatient care.^29-33^ International research has identified several key factors contributing to HT’s success: care delivered in the home environment, team mobility and flexibility, multiprofessional staffing, a broad range of interventions, access to inpatient beds when necessary, daily team contact, 24/7 team availability, and active involvement of the patient’s family and social network.^23^

## Objectives

### The First Home Care Program in Addiction Medicine

Despite these advantages, home treatment has not yet been systematically implemented in addiction medicine. Early HT models in both general and adolescent psychiatry frequently excluded individuals with substance use disorders.^34,35^ This omission is paradoxical, as addiction-focused interventions delivered within the very environment where substance use occurs may enable more contextually responsive, realistic, and ultimately more effective care.^36^

Recently, Längle^37^ proposed adapting the German model of “*stationsäquivalente Behandlung (StäB)”—*a home-equivalent psychiatric treatment framework*—*to patients with substance use disorders. In doing so, he also emphasized several specific challenges, including managing relapse risks in patients’ home environments, addressing fluctuating motivation, and ensuring effective coordination between addiction and psychiatric service. Adapting home treatment (HT) principles to addiction care, particularly for individuals with dual diagnoses, could therefore address a significant service gap and enhance continuity of care for this vulnerable population. To our knowledge, such an approach has not yet been implemented, neither in Switzerland nor elsewhere.

Even before Längle’s editorial appeared, the addiction service at the University Hospitals of Geneva (HUG) had decided to implement home treatment (HT), drawing on existing HT programs in general adult psychiatry in Switzerland as a model. The Geneva HT model for addiction care was introduced in 2022 to address local needs in a catchment area of approximately 530 000 inhabitants, served by 20 inpatient addiction treatment beds but with long-standing expertise in community-based approaches.^12,13,38^

The present study aimed to evaluate the feasibility and safety of implementing HT for patients with alcohol and substance use disorders (AUD/SUD) within a tertiary psychiatric care setting. We hypothesized that HT would be feasible for the majority of eligible patients, characterized by high completion rates and a low incidence of adverse events (safety). Furthermore, we anticipated that preliminary outcome indicators*—*treatment duration, clinical improvement, and service utilization*—*would be comparable to, or better than, those observed among matched patients receiving inpatient treatment (IT).

## Methods

### Study Design, Setting, Participants, and Power Consideration

The trial employed a retrospectively matched, 2-arm, parallel-group design comparing home treatment (HT) with inpatient treatment (IT) to evaluate the new HT program. The program was launched exclusively for individuals with AUD and SUD (ICD 10: F1x) in the context of dual diagnoses (F1x plus F2x-F7x), and its framework was aligned with the HT criteria for general psychiatric patients as defined by Gühne et al.^23^ The structure and components of the Geneva HT program have been described in detail elsewhere.^14,15^

Briefly, eligibility required a clinical indication for inpatient addiction treatment as assessed by the referring psychiatrist, addiction specialist, or primary care physician

Exclusion criteria included involuntary admission, absence of a permanent residence, history of complicated withdrawal, and acute suicidality. All patients requesting hospital admission participated in a preadmission interview to discuss the option of HT versus conventional inpatient care.

The HT program comprised up to 3 daily nurse visits, 7 days a week, and 1 to 2 physician visits per week, over an average duration of 2 to 3 weeks. A social worker was available upon request. Psychiatric and somatic assessments, as well as the therapeutic interventions described below, were provided as part of the program. The staff was identical with parts of the trained staff on the ward. Out of 9 potential care providers*—*5 nurses, 3 residents, and 1 senior physician*—*the number of active team members varied according to the number of patients enrolled in HT. A social worker was available on request and a consultant supervised the team.

Over the course of the year 2023, 60 requests for HT were received, of which 41 individuals were deemed eligible. Eligibility criteria included undergoing HT for the first time (repeated requests were possible) and not participating in another study. Prior to HT initiation, 1 individual withdrew consent and another required admission to a somatic hospital. Consequently, data from all remaining 39 eligible individuals were included in the study.

Inpatient treatment (IT) on a 20 bed ward (+2 extra beds) similarly involved the management of acute withdrawal symptoms and the treatment of co-occurring psychiatric and somatic conditions. Both therapeutic programs are multimodal, integrating dual diagnosis treatment with elements of psychoeducation, self-harm reduction, and motivational interviewing. The individually tailored treatment plan centered around future life projects and did not necessarily include abstinence as a goal.

Start and end dates for both HT and IT were defined as the dates of admission and discharge, respectively. The 1-year follow-up period was set as 365 days post-discharge, during which emergency room visits and hospitalizations were collected to provide an overview of patient outcomes. If follow-up for addiction treatment took place in a doctor’s office as opposed to the HUG outpatient clinics, this part of the treatment was not accessible to us. We thus did not include regular outpatient care.

Power considerations: After retrospective data collection, we performed a post-hoc sensitivity power analysis using G*Power 3.1^39^ for an independent-samples *t*-test (2-tailed, α = .05). For the available sample (HT n = 39; IT n = 43), the analysis indicated that the study had 80% power to detect between-group differences of at least *d* = 0.62 (Cohen’s *d*), corresponding to effects in the small range. Given the available sample size, the study was only powered to detect medium-to-large effects (*d* ≥ 0.62) and was underpowered to detect small effects (e.g., *d* = 0.20).

### Data Sources, Variables, Bias

We extracted all relevant data from electronic health records, including sociodemographic variables and diagnostic information. Addiction and general psychiatric diagnoses were recorded according to ICD-10, following institutional requirements. The Health of the Nation Outcome Scale (HoNOS)^40^ scores*—*routinely completed for each patient at both admission and discharge*—*were also obtained. Somatic conditions were included if they required hospitalization (e.g., cancer, trauma, pancreatitis) or represented chronic illnesses with a significant impact on daily functioning (e.g., liver disease, cardiovascular disease, COPD). These conditions were further classified according to whether they were direct sequelae of substance use or represented unrelated medical comorbidities.

Moreover, follow-up data were obtained from electronic medical records, which capture all hospitalizations and emergency department visits and therefore constitute a comprehensive source of clinical information.

This comprehensive follow-up is possible in Geneva because the HUG are the sole providers of acute psychiatric and somatic hospital care in the canton, and, due to insurance regulations, patients cannot seek treatment outside the canton. Although private somatic hospitals exist, none of the patients included in this study had private health insurance.

The main source of bias stems from clinical judgment, which may vary between physicians. This primarily affects the prioritization (hierarchization) of diagnoses. To minimize this bias, we reported all diagnoses irrespective of their assigned priority. Similarly, variability may occur in the rating of HoNOS items due to subjective interpretation.

Several measures were implemented to reduce these biases. First, all patients’ diagnoses were systematically discussed with a senior physician. Second, rater training sessions for the HoNOS scale were conducted for newly arriving interns. After each patient interview and individual rating, the case was discussed with a senior physician. The procedure was repeated across several patient admissions until consensus was achieved. When necessary, additional sessions were conducted to ensure a stable agreement.

### Matching Rationale

Matching was conducted based on age and gender to minimize confounding by demographic factors that could influence service utilization. Age and gender are well-established predictors of psychiatric hospitalization and emergency service use. Due to the pilot nature of this study and the limited sample size, matching was restricted to these core variables to maintain stable group sizes and prevent overfitting. Given these constraints, we prioritized exact matching on key demographic variables rather than propensity score matching, which would likely have resulted in substantial data loss. Covariate balance was assessed using standardized mean differences (SMDs), with values below 0.1 indicating adequate balance.

### Outcome

The primary outcome was defined as feasibility and safety. Feasibility was measured by treatment completion versus withdrawal, while safety was operationalized as the need for urgent hospital admission due to any crisis during HT. Secondary outcome measures included discontinuation of index treatment and service utilization parameters during the 12-month follow-up period. Service utilization was defined as the number of emergency department visits and the number of hospitalization days, categorized into admissions for addiction-related reasons, substance-related sequelae, and somatic conditions.

### Ethical Considerations

All patients provided written informed consent for treatment and for the use of their anonymized health data during the admission process. The study relied exclusively on routinely collected anonymized hospital data and therefore did not require approval from the local ethics committee. Anonymized data were destroyed upon completion of the dataset. This approach is in accordance with Geneva’s cantonal regulations, including directives from the ethics committee, as well as Swiss law, which permits the use of anonymized data for research purposes when general consent has been provided at admission. All procedures adhered to the principles of the Declaration of Helsinki.

The study was not registered.

The STROBE checklist^41^ was used as a framework for reporting observational studies (see Table 4, supplementary material).

### Statistical Analyses

Statistical analyses were conducted using IBM SPSS Statistics (version 27).^42^ Descriptive statistics were used to summarize demographic and clinical characteristics. The Kolmogorov–Smirnov test was employed to assess the normal distribution variables.

Depending on the level of measurement and distribution of the variables, independent-samples t-tests, chi-square/Fisher’s exact tests, or Mann–Whitney U tests were used to compare groups.

Effect sizes, including standardized mean differences, were calculated both to assess baseline covariate balance and to compare outcome measures. Effect sizes were used to quantify the magnitude of differences or associations between the HT and IT groups, independent of sample size.

Missing data: HoNOS admission scores were missing for 2 participants in the HT group. Given the low rate of missingness (<5%) and the absence of systematic patterns, complete case analysis was performed. No imputation was applied.

## Results

All inpatients (IT) treated in 2023 were screened and matched to the 39 HT patients, based on age and gender. This resulted in a comparison group of 43 IT patients.

### Matching

The data on age and gender are given in Table 1. Effect sizes for both variables are negligible to very small, which indicates that the groups do almost not differ as was foreseen by the matching process.

**Table 1.** Sociodemographic Variables of Patients in Home Treatment (HT) and Inpatient Treatment (IT).

Variable	Test	HT n = 39	IT n = 43	Level of significance *t* or *P* exact, 2-tailed	Effect size Cohen’s *d*, 95% CI or Cramer’s V	Interpretation
Age years mean (SD)	*t*-test	47.4 (12.2)	48.0 (10.9)	*t* = −0.491	*d* = 0.11, 95% CI (−0.54, 0.33)	(Very) small effect
Gender male:female	Fisher’s exact	19:20	21:22	*P* = 1.000	V = 0.01	Negligible effect
Curatorship no:yes	Fisher’s exact	12:27	14:29	*P* = 1.000	V = 0.019	Negligible effect
Partnership						
Single = 1, couple = 2, shared apartment = 3, separated, divorced, widowed = 4	Fisher’s exact	1:2:3:4 12:11:5:11	1:2:3:4 21:11:1:10	*P* = .179	V = 0.247	Small to medium effect
Housing						
Independent = 1, family = 2 institution/hotel** = 3	Fisher’s exact	1:2:3 36:3:0	1:2:3 34:5:4	*P* = .150	V = 0.231	Small to medium effect
Employment						
Employment = 1, unemployment = 2 pension, sick leave, disability = 3 social welfare = 4 other = 5	Fisher’s exact	1:2:3:4:5 9:6:19:5:0	1:2:3:4:5 5:6:18:8:6	*P* = .012	V = 0.306	Medium effect

Abbreviation: CI, confidence interval.

Values are presented as mean (SD) or counts.

*t*-test and Fisher’s exact test were used with respect to the variables’ scale level, the indicators of the effect sizes depend on the tests, that is, Cohen’s d refers to the t-test and Cramer’s V to Fisher’s exact test.

* Couple = any type of stable intimate relationship (marriage, registered partnership, or other).

** Includes hotel rooms, a Geneva speciality.

### Sociodemographic Variables

Sociodemographic variables for both groups are also presented in Table 1, including housing situation, employment, partnership, and curator status.

Curatorship does not differ between the groups, whereas partnership and housing showed a slight and employment a moderate imbalance between groups—the HT groups comprised less individuals who were single, more who were employed and no one in an institution or hotel, although HT can be implemented at these places.

### Clinical Variables

Table 2 presents the distribution of diagnoses across categories. The maximum number of diagnoses per individual, regardless of type, was 6; this upper limit was not reached in the HT group for any diagnosis category. Regarding addiction diagnoses, the HT group clearly exhibited a smaller total number of diagnoses. For psychiatric and somatic diagnoses, the pattern points in a similar direction, although less markedly. Overall, severe somatic diagnoses were widely present, severe was defined as, for example, pronounced liver, lung or heart disease, pancreatitis, cancer.

**Table 2.** Clinical Variables of Patients in Home Treatment (HT) and Inpatient Treatment (IT).

Variable	Test	HT n = 39	IT n = 43	Level of Significance *t* or *P exact*	Effect size Cohen’s *d*, 95% CI or Cramer’s V	Interpretation effect size
Number of addiction diagnoses 1-6	Fisher’s exact	1:2:3:4:5:6 17:10:3:7:2:0	1:2:3:4:5:6 8:13:4:10:7:1	*P* = .146	V = 0.310	Medium
Number of psychiatric diagnoses 0-6	Fisher’s exact	0:1:2:3:4:5:6 3:12:11:7:4:2:0	0:1:2:3:4:5:6 0:17:14:5:5:1:1	*P* = .506	V = 0.266	Small to medium
Number of somatic diagnoses 0-6	Fisher’s exact	0:1:2:3:4:5:6 3:12:11:7:4:2:0	0:1:2:3:4:5:6 0:17:14:5:5:1:1	*P* = 0.483	V = 0.266	Small to medium
Duration of index hospitalization	*t*-test	14.7 + 4.6	16.6 + 12.5	*t* = −0.900	*d*: −0.199 95% CI (−0.63; 0.24)	Small
HoNOS admission	*t*-test	18.5 + 6.6*	18.4 + 7.3	*t* = −0.126	*d*: −0.028 95% CI (−0.46, 0.41)	None
HoNOS discharge	*t*-test	11.2 + 7.2	11.1 + 5.2	*t* = 0.055	*d*: 0.012 95% CI (−0.43, 0.45)	None
Discharge on discontinuation no: yes	Fisher’s exact	39:0	32:11	*P* = .001	V = 0.375	Medium

Abbreviations: CI, confidence interval; HoNOS, Health of the Nations Outcome Scale.

Values are presented as mean (SD) or counts.

* n = 37.

With respect to dual diagnoses, 3 patients in the HT group did not have a co-occurring psychiatric diagnosis, representing 3.7% of the total sample.

The duration of index hospitalizations was relatively comparable between the 2 groups. HoNOS ratings, which reflect disease severity and functional impairment, did not differ at admission and at discharge, and both groups showed improvement over the course of treatment. It should be noted that 2 admission HoNOS ratings were missing in the HT group. Admission HoNOS scores did not differ between HT and IT groups, suggesting that missing data were unlikely to bias group comparability.

While there were no treatment cessations or transfers to inpatient care among HT patients, approximately 25% of patients in the IT group discontinued treatment*—*representing a clear difference between the groups.

As suicidality and a history of complicated withdrawal were exclusion criteria for HT, both conditions were expected to occur only in the IT group. The ratios were 0:7 for complicated withdrawal and 2:14 for suicidality (HT:IT). Suicidality was not anticipated in the HT group; however, 1 patient was admitted due to special circumstances, and another was included because her history was not known*—*she had explicitly concealed this information, and it was not documented in her medical records.

**Figure 1.**
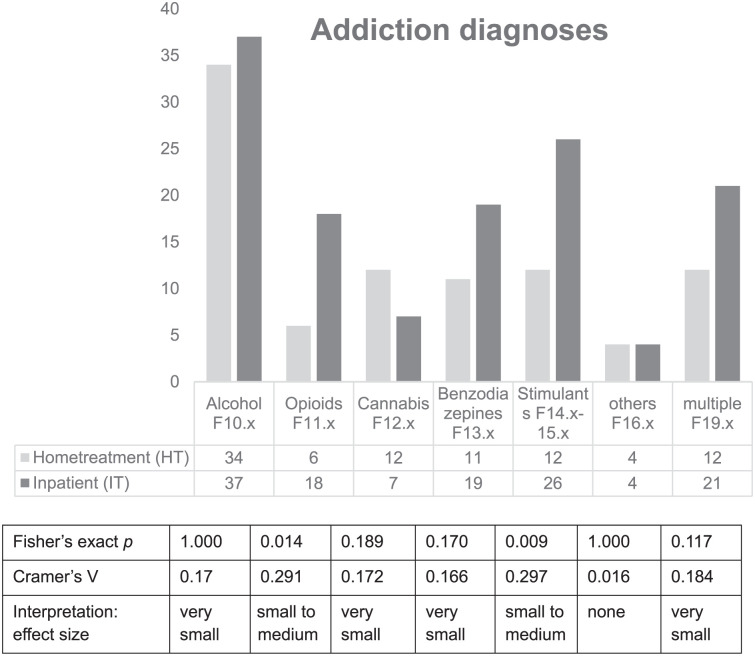
The different addiction diagnoses and their distribution among HT and IT F-numbers refer to ICD-10 chapters.

Figure 1 depicts the distribution of addiction diagnoses in both groups. The most common diagnoses was alcohol dependence with a small difference between groups. Similarly this pattern arises for cannabis, benzodiazepines, and multiple drug use. Other substances such as ketamine and pregabalin did hardly play any role in both groups.

However, differences arose with respect to opioids and stimulants, both were substances more frequently used in the IT group.

**Figure 2.**
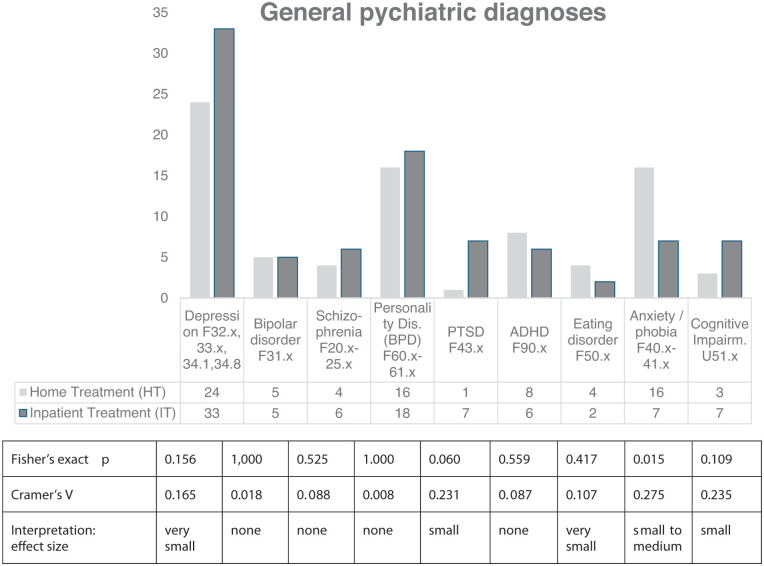
General psychiatric diagnoses of Home Treatment patients (HT) and Inpatients (IT) F numbers refer to the ICD-chapters.

As given in Figure 2, a variety of diagnoses from general psychiatry was present in both groups. Overall depression, personality disorders, and anxiety were frequent. Groups were comparable with respect to bipolar disorder, schizophrenia, personality disorders, and ADHD.

Only slight differences arose for depression, being more prevalent in the IT group, and for eating disorders with a tendency towards the HT group. Cognitive impairments and PTSD were somewhat higher in the IT group, whereas a clear difference arose regarding anxieties and phobia in HT.

### Service Use During 1 Year Following Treatment

Table 3 gives service use during the year after index treatment, that is, up to 365 days after discharge. Whereas hospitalizations for addiction differ slightly, there right-skewedness varies remarkably, it is moderately present in HT and strongly so in IT.

**Table 3.** Service Use During 1 Year Following Index Hospitalization.

Variable	Test	HT n = 39 median, range	IT n = 43 median, range	test statistic	Standardized test statistic	Level of significance	Effect size *r*	Interpretation effect size
Hospitalization for addiction	Mann-Whitney-*U*	12.00 (0-114)	1.00 (0-149)	*U* = 725.50	*Z* = −1.093	*p* = 0.275	*r* = −.121	Small
Hospitalization for sequels	Mann-Whitney-*U*	0.00 (0-25)	0.00 (0-193)	*U* = 897.00	*Z* = 0.650	*p* = 0.516	*r* = .072	Negligible
Hospitalization-independent	Mann-Whitney-*U*	0.00 (0-46)	0.00 (0-59)	*U* = 867.00	*Z* = 0.313	*p* = 0.754	*r* = .035	Negligible
Emergency room visits	Mann-Whitney-*U*	1.00 (0-16)	2.00 (0-14)	*U* = 957.00	*Z* = 1.120	*p* = 0.263	*r* = .124	Small

With respect to hospitalizations for sequels and other, independent reasons, the groups are almost similar. Regarding emergency room visits a small difference arose as the IT group presented with emergencies more often.

Additional material Table 5 shows service use during the year prior to the index treatment. Hospitalizations for addiction and for sequels as well as emergency room visits were comparable between groups, hospitalizations for somatic purposes was not assessed.

## Discussion

This study retrospectively presents the first results of HT for individuals with AUD/SUD—a group that, despite their severity of illness and psychiatric as well as somatic co-morbidity, high service utilization, and frequent hospital readmissions, has been systematically excluded from existing HT.^1,34,35^ At the same time community services have become standard in general psychiatry in Europe and worldwide. However, to our knowledge, only a few studies did not completely exclude addiction, Bechdolf et al^20^ give a participation rate of 6.5% and Weinmann et al^27^ a rate of 24% in HT.

The present study addresses this gap by using principles already established in general psychiatry.^23,43,44^ by focusing on patients with addiction only. All patients which were possibly available from the first year of ongoing, consecutive HT were explored and matched with inpatient. HT stood for itself and fully replaced inpatient treatment.

### Outcome

The main finding of this study is that HT for addiction patients with dual diagnoses was feasible and safe: all patients completed the planned treatment period without requiring urgent hospitalization. This result is consistent with findings from HT in general psychiatry, as reported in several recent*—*partly large*—*studies.^20,22,30,45-47^

A secondary outcome was treatment discontinuation. In our HT group, no patient discontinued treatment, whereas in the IT group approximately 25% of patients terminated treatment prematurely*—*either by leaving the open ward unannounced or by being discharged due to recurrent inappropriate behaviors such as aggression or dealing. Treatment discontinuation is common in general psychiatry and even more so in addiction care. Notably, in contrast to our findings, Weinmann et al^27^ observed discontinuation rates of 7% in HT and 10.5% in IT.

Further secondary outcome measures were comparable between groups: hospitalization days*—*whether for addiction-related reasons, psychiatric sequelae, or other somatic conditions*—*and the use of emergency department services. Some studies in general psychiatry report lower inpatient days during following in the HT group.^20,22,27,30^ Hospitalizations for sequelae or somatic causes are generally not reported in these studies.

Regarding emergency room visits, no relevant data were identified in the available HT literature. This may indicate that ED use is either a less prominent issue in other settings or simply not routinely captured as an outcome.

Apart from primary and secondary outcome measures, several additional variables merit attention. On the dual diagnosis realm, this patient sample presents with comorbid rates similar to general psychiatric patients without AUD/SUD.^48-53^

The length of treatment should also be considered. Across the international literature on general psychiatric patients, HT duration tends to be longer than in our sample. In the United Kingdom, treatment episodes usually last 1 to 3 weeks.^54,55^ In Scandinavia,^16^ the typical duration is of about 2 to 4 weeks. Swiss studies report a duration of up to 5 and 6 weeks.^30,56^ In Germany, HT commonly lasts about 4 to 6 weeks,^20,27^ while in the Netherlands durations between 4 and 8 weeks are described.^22^

Sociodemographic difficulties, especially unemployment represent a problem in all areas of psychiatry, thus our patient group does not differ.^57,58^ A number of studies have shown that all of the above-mentioned factors improved through outreach treatment (overview^59^).

### Limitations

This study has several limitations.

First, treatment allocation was not randomized. During matching, small age discrepancies had to be accepted.

Second, the study was conducted retrospectively.

Third, the study was underpowered. The relatively small sample size and the retrospective design reduce the generalizability of the findings. Although the matching process improved group comparability, unmeasured confounders such as unreported consumption cannot be ruled out, raising*—*among other issues*—*the question of whether diagnostic distributions were accurately captured.

Forth, documentation inconsistencies and the limited integration of hospital and ambulatory records may have affected data completeness and diagnostic accuracy. This was addressed through extensive manual data extraction. Moreover, the equivalence of several key variables (e.g., HoNOS scores, comorbidities) suggests that core assessments were conducted with similar rigor in both groups.

Fifth, observed differences in exclusion criteria reflect the deliberate structure of the program and limit the generalizability of the findings to a broader AUD/SUD population.

Sixth, we did not assess patient satisfaction, quality of life, or the perspectives of relatives.

Seventh, comparisons had to be made against the HT literature covering general psychiatry as research on this topic has not been executed.

### Future Directions

Most importantly, HT for patients with dual diagnoses should become an integral part of the Swiss healthcare system. Until now, HT has been implemented and delivered in addition, with the regular resources of an inpatient ward, rather than being structurally anchored and sustainably financed.

This study provides an empirical foundation for further research on HT in addiction medicine. Robust evidence will require larger samples and randomized study designs. Future investigations should evaluate long-term outcomes such as abstinence, controlled consumption, relapse rates, patient-reported outcomes, patient and family satisfaction, quality of life, and cost-effectiveness. The active components of HT*—*such as treatment frequency, network involvement, crisis management, and other team features (e.g., crisis intervention capacity)*—*should also be examined to determine which elements contribute most to treatment success. In addition, qualitative studies could explore the perspectives of patients, relatives, and clinicians to better understand acceptability, barriers to implementation, and opportunities for program refinement.

## Conclusions

Our findings indicate that home treatment for patients with AUD/SUD is both feasible and safe when guided by established principles from general psychiatry and when exclusion criteria are clearly defined. Given the chronic and complex nature of addiction, care models that operate within the patient’s living environment and actively involve their social context are essential. Home treatment in addiction psychiatry may represent a promising step toward a more flexible, responsive, and integrated model of care.

## Supplemental Material

sj-docx-1-jpc-10.1177_21501319251412650 – Supplemental material for Hospital-at-Home for Alcohol and Substance Use Disorders Compared to Inpatient Treatment in Dual Diagnosis Patients: A Retrospectively Matched Cohort Pilot Study Incorporating Service Use 12 months Pre- and Post-Treatment in Geneva Supplemental material, sj-docx-1-jpc-10.1177_21501319251412650 for Hospital-at-Home for Alcohol and Substance Use Disorders Compared to Inpatient Treatment in Dual Diagnosis Patients: A Retrospectively Matched Cohort Pilot Study Incorporating Service Use 12 months Pre- and Post-Treatment in Geneva by Silke Bachmann, Pantelis Baniotopoulos, Corinne Amoros, Louise E. Penzenstadler and Daniele F. Zullino in Journal of Primary Care & Community Health

sj-docx-2-jpc-10.1177_21501319251412650 – Supplemental material for Hospital-at-Home for Alcohol and Substance Use Disorders Compared to Inpatient Treatment in Dual Diagnosis Patients: A Retrospectively Matched Cohort Pilot Study Incorporating Service Use 12 months Pre- and Post-Treatment in Geneva Supplemental material, sj-docx-2-jpc-10.1177_21501319251412650 for Hospital-at-Home for Alcohol and Substance Use Disorders Compared to Inpatient Treatment in Dual Diagnosis Patients: A Retrospectively Matched Cohort Pilot Study Incorporating Service Use 12 months Pre- and Post-Treatment in Geneva by Silke Bachmann, Pantelis Baniotopoulos, Corinne Amoros, Louise E. Penzenstadler and Daniele F. Zullino in Journal of Primary Care & Community Health
