# Supplementary material for: Hospital-at-Home for Alcohol and Substance Use Disorders Compared to Inpatient Treatment in Dual Diagnosis Patients: A Retrospectively Matched Cohort Pilot Study Incorporating Service Use 12 months Pre- and Post-Treatment in Geneva
Source: J Prim Care Community Health. 2026 Mar 31;17:21501319251412650. doi: 10.1177/21501319251412650 (PMC13039575; doi:10.1177/21501319251412650)
Supplement: sj-docx-2-jpc-10.1177_21501319251412650 – Supplemental material for Hospital-at-Home for Alcohol and Substance Use Disorders Compared to Inpatient Treatment in Dual Diagnosis Patients: A Retrospectively Matched Cohort Pilot Study Incorporating Service Use 12 months Pre- and Post-Treatment in Geneva [file sj-docx-2-jpc-10.1177_21501319251412650.docx]

Table 5: Service use during 1 year prior to index hospitalization

| Variable | Test | HT  n = 39  median, range | IT  n = 43  median, range | test statistic | Standardized test statistic | Level of Significance | Effect  size  r | Interpretation  effect size |
| --- | --- | --- | --- | --- | --- | --- | --- | --- |
| Hospitalization  for addiction | Mann-Whitney-U | 0.00  (0-153) | 0.00  (0-197) | U = 972.00 | Z= 1.332 | *p* = 0.183 | r= 0.147 | small |
| Hospitalization for sequels | Mann-Whitney-U | 0.00 (0-56) | 0.00  (0-71) | U = 852.50 | Z = 0.179 | *p* = 0.858 | r= 0.020 | none |
| Emergency Room Visits | Mann-Whitney-U | 0.00  (0-16) | 1.00 (0-16) | U = 927.50 | Z = 0.882 | *p* = 0.378 | r= 0.097 | small |
